# Post-Operative Hypertension after Total Knee Arthroplasty and the Effects on Transfusion Rates

**DOI:** 10.1371/journal.pone.0050967

**Published:** 2012-12-26

**Authors:** Russell R. Russo, Vinod Dasa, Robert Duarte, Burton Beakley, Manish Mishra, Hilary Thompson

**Affiliations:** 1 Department of Orthopedic Surgery, Louisiana State University Health Sciences Center, New Orleans, Louisiana, United States of America; 2 School of Medicine, Louisiana State University Health Sciences Center, Shreveport, Louisiana, United States of America; 3 Tulane School of Medicine, New Orleans, Louisiana, United States of America; 4 Department of Biostatistics, Louisiana State University Health Sciences Center, New Orleans, Louisiana, United States of America; University of Colorado, United States of America

## Abstract

Transfusions are a cause of significant patient morbidity as well as expense. Anesthesia literature has examined controlled intraoperative hypotension as a means for reducing blood loss and transfusions. Our hypothesis is that inversely increased blood pressure post-operatively would then lead to increased blood loss and transfusions.

We examined 105 consecutive patients who underwent TKA. We found a significant odds ratio of 1.123 for pre-operative hematocrit. For post-operative blood pressure, we calculated an insignificant odds ratio of 1.007, proving no relationship between post-operative blood pressure and transfusions.

This is the first study to examine increased post-operative blood pressure's contribution to transfusion rates. Although we confirmed that low pre-operative hematocrit contributes to increased transfusions, we did not find a relationship between post-operative blood pressure and transfusions.

## Introduction

Total joint replacements, in particular primary total knee replacements, has tripled in recent years [1]. With this rapid increase in total knee arthroplasties on an increasingly older population, there has been a concurrent rise in complications as well a rise in allogeneic blood transfusions [2]. Blood transfusions after unilateral total knee replacements have been reported to be 20% [3].

Allogenic blood transfusions have significant risks and a significant financial burden, estimated to be between $522–1,183 per unit [4]. Also allogeneic blood transfusions can lead to a variety of significant medical problems including transfusion reaction, infection, and even the rare transmission of HIV and Hepatitis [5].

To prevent the associated morbidity and cost of transfusions, one must closely monitor both intraoperative and postoperative blood loss. Average intraoperative blood loss ranges between 800–1200 milliliters per case with varied postoperative blood loss via wound drainage or drain outputs [6]. Despite advances in total knee arthroplasty including cementless components, navigation, new pre-operative nutritional protocols, and fibrin sealants, the procedure still entails significant blood loss. This blood loss leads to the need for transfusions [6]. Several variables have not been found to have a connection to transfusions including patient age, sex, BMI, pre-operative blood pressure, and use of epinephrine in the wound prior to closure [7].

One practical method employed during total joint procedures is controlled hypotension. During a procedure, the rise and fall of a patient's blood pressure is evident to the surgeon by the amount of generalized bleeding from an exposed operative bed. This is the basis behind controlled intraoperative hypotension employed by the anesthesia team as a means to reduce intraoperative blood loss [8], [9]. In addition to bleeding intraoperatively, patients continue to bleed postoperatively with up to 50% of total postoperative blood loss within the first 3 hours after surgery and 80% within the first 24 hours [9].

With a significant amount of a patient's total blood loss occurring after the procedure, one should examine post-operative variables in order to establish ways to mitigate blood loss and thus transfusion rates. Our hypothesis is that post-operative hypertension is related to postoperative blood loss and subsequently the risk of transfusions.

## Methods

Prior to our review of patient data, the Institutional Review Board for both Ochsner Medical Center-Kenner and LSUHSC-New Orleans approved this retrospective chart review. A waiver of consent was also obtained from both IRBs as this retrospective review did not link any patient identifiers to our data. Information was kept stored in security and password protected computer storage system.

Based on similar recent studies, a retrospective chart review of 105 consecutive primary total knee arthroplasties performed between March of 2009 and May of 2010 at a single facility by a single surgeon was performed [7]. All patients who underwent primary total knee arthroplasty were included. Patients who had history of bleeding disorders, who were on pre-operative anticoagulation, who refused blood transfusion, or who required revision arthroplasty were excluded from the review.

The same surgical technique was employed for each case. A nonsterile tourniquet was placed and inflated on the proximal thigh prior to incision. A standard midline anterior approach with a medial parapatellar arthrotomy was used. Stryker total knee navigation was employed for both femoral and tibial cuts. Appropriate components were placed including a cemented femoral component and either a cementless monoblock component or standard cemented tibial component. The patella was resurfaced in 54% of patients. The tourniquet remained inflated only to be deflated briefly to check for bleeding in the posterior capsule after all bony cuts were complete and then re-inflated once again. A bovie electro cautery device was used throughout each case as well where indicated. No fibrin sealants or other intraoperative hemostasis products were used.

A postoperative medium hemovac drain was placed and cut between the 7^th^ and 8^th^ hole to assure equal chance for drain output amongst all patients. The drain was pulled on all patients within 24 hours of the procedure. Patients were started on Lovenox 30 mg twice a day subcutaneously for DVT prophylaxis along with TED stockings and Sequential compressions devices.

Data including patient age, sex, BMI, pre and post-operative hemoglobin and hematocrit, blood pressure, drain output, and transfusion were analyzed. All data points were obtained from anesthesia, recovery room, and inpatient hospital records. Our criteria for transfusion remained constant with transfusions triggered by hemoglobin and hematocrit equal or less than 8/24 (Hgb/Hct) or patients symptomatic with tachycardia, dizziness, or lightheadedness with Hgb less than or equal to 10 who did not respond to an initial fluid bolus of normal saline.

### Statistical Analysis

The analysis of the variables collected on the retrospective patient series were considered as multiple risk factors in a multivariate logistic regression model [10]. The outcome variable in the analysis was the need for transfusion expressed as a binary variable (0 = transfusion not needed, 1 = transfusion). Risk factors evaluated in stepwise variable selection procedures were all variables considered as potential risk factors for surgeries requiring transfusion (see list above and in results). Overall statistical significance of the multiple logistic regression models was evaluated by the Hosmer-Lemeshow goodness of fit test. Significance of individual variables as significant risk factors was evaluated by a significant Chi-square probability for inclusion of the variable in the final model after stepwise variable selection and by the width and non-inclusion of the value 1 in the confidence intervals for the odds ratios. All data manipulation, management and analysis were carried out using procedures of and programs written in the Statistical Analysis System language (SAS Institute, Cary NC).

## Results

The charts of 105 consecutive patients between the ages of 36–89 were retrospectively analyzed. Several variables were recorded including the patient's age, BMI, blood pressures both in the recovery room and on the floor, intraoperative blood loss, hemovac drain output (recovery room and inpatient floor), and pre-operative hematocrit. Of the 105 studied patients 40 (38%) required blood transfusion according to the strict guidelines reviewed above in the methods section.

The average age of the non-transfusion group (n = 65) was 65.6 years old (range 36–89) while the transfusion group (n = 40) had a similar average age of 65.7 years old (range 42–87). Similarly, the BMI average between the groups were comparable with 34.8 (range 23–53) for the non-transfusion group and 33.2 (range 22–58) for the transfusion group. Blood pressures for both cohorts were comparable as well. Recovery room and floor average blood pressures for the non-transfusion group were 135/74 mmHg and 143/78 mmHg, respectively, while the results for the transfusion group were 129/72 mmHg and 141/74 mmHg. All of the variables averages, including ranges and standard deviations, can be viewed in *Table 1* where all are included.

**Table 1:** Variable Means in Cohorts.

	Non-Transfusion (range)	Transfusion (range)
Age	**65.6** (36–89) std: 10.7	**65.7** (42–87) std: 10.4
BMI	**34.8** (23–53) std: 6.0	**33.2** (22–58) std: 7.8
Systolic (Recovery)	**134.9** (96–199) std: 22.9	**128.8** (91–170) std:22.7
Diastolic (Recovery)	**74.2** (46–96) std: 11.4	**72.3** (45–100) std: 13.5
Systolic (Floor)	**143.5** (112–204) std: 20.4	**140.6** (97–197) std: 24.6
Diastolic (Floor)	**77.6** (43–102) std: 11.7	**74.4** (54–98) std: 10.5
Drain output (Recovery)	**189.5** (0–800) std: 166.2	**195.1** (0–525) std: 127.2
Drain output (Floor)	**321.7** (0–1900) std: 303.6	**332.3** (0–1000) std: 259.0
Operative Blood Loss	**129** (0–300) std: 79.6	**149.4** (0–750) std: 148.6
Pre-Operative HCT	**35.5** (25–42.6) std: 4.4	**32.1** (20.5–45.8) std: 5.5

Other variables tested included intraoperative blood loss and cumulative hemovac drain outputs. Intraoperative blood loss averaged 129cc in the non-transfusion group vs. 149cc in the transfusion group however the difference was not statistically significant. The Recovery room and floor hemovac cumulative drain outputs for the non-transfusion group was 189.5cc and 321.7cc respectively versus the transfusion group whose outputs were 195.1cc and 332.3cc. All drains were removed the next morning about 24 hours following surgical start time. Despite a noticeable difference the averages were also not statistically significant (p-value<.05). Lastly, pre-operative hematocrit of the non-transfusion versus transfusion group was 35.5 to 32.1 respectively, which was a significant difference (p-value<.05).

From this analysis, we calculated odds ratios for each individual variable in order to delineate if each variable had a positive or negative effect on the patient requiring transfusion. The odds ratio was calculated to determine which parameters would lead to a significant increase in transfusions. Only one variable produced a statistically significant value and that was of pre-operative hematocrit which had an odds ratio of 1.123 (95% confidence interval 1.020–1.236), implying a significant relationship between a patient's pre-operative hematocrit and the need for transfusion. However, the odds ratio for post-operative systolic blood pressure was 1.007 (95% confidence interval.986–1.029), suggesting there is no significant relationship between post-operative hypertension and the need for transfusion which was the focus of this investigation. Odds ratios for all other variables including BMI, blood pressures, intraoperative blood loss, hemovac outputs can be reviewed in *Table 2*. These other variables did not produce any statistically significant odds ratios and therefore are found to have neither a positive or negative influence on whether a patient requires allogeneic blood transfusion.

**Table 2:** Odds Ratios of Variables against Transfusion.

	Odds Ratios	95% Confidence Interval
BMI	1.065	(.988–1.148)
Systolic (Recovery)	1.008	(.964–1.054)
Diastolic (Recovery)	1.036	(.969–1.108)
Systolic (Floor)	1.007	(.986–1.029)
Drain output (Recovery)	1.000	(.994–1.006)
Drain output (Floor)	1.001	(.998–1.004)
Operative Blood Loss	.995	(.988–1.002)
Pre-Operative HCT	1.123	(1.020–1.236)**

** Statistically significant as 1.000 not included in confidence interval.

## Discussion

This study is the first of its kind to investigate a relationship between postoperative blood pressure and a patients' total blood loss and need for transfusion. Although results show no statistically significant correlation between postoperative hypertension and blood loss, it helps in directing further efforts and research towards controlled hypotension intraoperatively.

Although the results did not indicate a relationship with hypertension post-operatively, it did confirm previous study results that a main determinant for transfusion is pre-operative hematocrit levels [5], [7]. All other data collection points such as age, sex, BMI did not show a causal relationship with transfusion rates also confirming previous studies findings [7].

With the continued verification of pre-operative hemoglobin, hematocrit, and erythrocyte count as a risk factor for post-operative transfusions, it seems more emphasis should be placed on controlling these levels. However, studies have shown that pre-operative autologous donation is neither cost effective nor beneficial [11]. Therefore, recent regimens of iron, folic acid, vitamin C have been proposed combined with erythropoietin. These studies have shown significant reduction in the need for allogeneic blood transfusion [12].

The associated morbidity of transfusions along with the associated financial burden has made preventing blood transfusions after total joint replacement a significant problem amongst orthopedic surgeons [4]. Although no relationship was identified with post-operative hypertension and transfusion rates, pre-operative hematocrit levels continue to be found to correlate with transfusion rates. This is where future research should be aimed in efforts to mitigate transfusion rates in total knee arthroplasty.
